# Advancements in surgical education: exploring animal and simulation models in fetal and neonatal surgery training

**DOI:** 10.3389/fped.2024.1402596

**Published:** 2024-06-03

**Authors:** Emily L. Davidson, Kristina L. Penniston, Walid A. Farhat

**Affiliations:** University of Wisconsin School of Medicine and Public Health, Madison, WI, United States

**Keywords:** surgical education, animal models, surgical simulation, fetal surgical training, neonatal surgical training

## Abstract

**Introduction:**

Surgical education is undergoing a transformation, moving away from traditional models towards more modern approaches that integrate experiential and didactic methods. This shift is particularly pertinent in the realm of fetal and neonatal surgery, where specialized training is crucial. Historical training methods, such as cadaveric dissection, have been prevalent for centuries, but newer innovations, including animal and non-animal simulation models, are gaining prominence. This manuscript aims to explore the use of both animal and non-animal models in surgical education, with a specific focus on fetal and neonatal surgery.

**Animal models:**

The use of animal models in surgical training has a long history, dating back to Halsted's introduction in 1889. These models, often utilizing large animals like swine and dogs, offer valuable insights into fetal and neonatal surgeries. They allow for the study of long-term outcomes and the simulation of various diseases and anomalies, providing essential training experiences not readily available in human surgeries. However, there are notable limitations, including anatomical and physiological differences from humans, ethical considerations, and substantial infrastructure and maintenance costs.

**Simulation models:**

Simulation-based training offers several benefits, including standardized and safe learning environments without risks to real patients. Bench models, using synthetic materials or non-living animal tissue, provide cost-effective options for skills development. Virtual reality and 3-D printing technologies further enhance simulation experiences, allowing for the replication of complex clinical scenarios and patient-specific anatomies. While these models offer significant advantages, they lack the complexity of biological systems found in animal models.

**Conclusion:**

In conclusion, both animal and non-animal simulation models play crucial roles in enhancing surgical education, particularly in fetal and neonatal surgery. While advancements in non-animal technologies are important for ethical reasons, the continued necessity of animal models in certain areas should be acknowledged. By responsibly integrating these models into training programs, surgical education can be further enriched while upholding ethical standards and ensuring optimal patient outcomes.

## Introduction

Surgical education is being transformed from the traditional apprenticeship model to a modern system that integrates cutting edge experiential and didactic teaching methods. Older surgical residency program models did not always consider that trainees learn at different paces and have variable learning needs. In recent years, the importance of teaching surgical skills has been refined. More emphasis is placed on patient safety, efficient use of operating room time, individual trainees' learning needs, and financial concerns. These and other factors have exerted pressure on surgical faculty to modify their approaches to include training that is more specific and applicable to the human setting. The need for innovation in this area is particularly critical in fetal and neonatal surgical training. The landscape of postgraduate medical education has changed dramatically over the last decade, and the conventional training model has undergone scrutiny and modifications. The mandate of the 80-h work-week, the introduction of integrated residency programs, and increased global awareness about patient safety, along with financial constraints, have spurred changes in educational practices. In addition, new technologies and increasingly complex procedures have changed where and how technical and procedural skills are taught. As a result, the need for highly-relevant animal models as well as simulation-based training has been embraced by the surgical community as a method to aid traditional learning.

The history of surgical training has historically relied heavily on cadaver surgery. Cadaveric dissection has been used as an educational surgical model for centuries. Many training programs across the country use fresh tissue dissection laboratories to provide a realistic surgical environment and facilitate the learning of surgical anatomy, operative exposures, operative techniques, and instrument handling. In the cadaveric surgical environment, learners may practice team training and crisis intervention scenarios as outlined in the American College of Surgeons Phase III Simulation Curriculum (ACS-AEI phase III) (1). A primary advantage of cadaveric simulation for surgical training is accuracy of anatomy, which provides a more realistic learning experience for learners (2, 3). It allows for the practice of high-acuity skills for which there may be limited alternative opportunities, enhancing learning in both technical and nontechnical skills (3, 4). Additionally, cadaveric simulation is effective for teaching procedural skills and has been found to be superior to bench-top simulators for certain surgical training activities (5). The use of cadavers for learning clinical procedures is a realistic (high-fidelity) simulation opportunity and a valuable tool for surgical education (2).

Despite the obvious benefits of cadaveric tissue dissection, there remain a few drawbacks. Like animal models, there are ethical and regulatory considerations in cadaveric surgical training. These are expensive and frequently difficult to procure. Additionally, while cadaveric simulation offers high fidelity in anatomy, the absence of blood flow, blood pressure, and tissue turgidity limits its effectiveness in replicating physiological conditions, which is crucial for clinical relevance and comprehensive training (6). Without blood flow, tissue viability is compromised, limiting the practice of surgical techniques that require a realistic tissue response (7). The absence of blood pressure further hinders the simulation of hemodynamic responses and the assessment of procedures that are influenced by blood pressure dynamics (8). To address these issues, Minneti et al. developed a novel perfusion cadaver model with life-like blood flow and blood pressure capabilities (9). Using this model, they created a curriculum incorporating real-world scenarios and simulated injuries to teach surgical skills and error management strategies to novice trainees. The use of perfusion techniques to restore blood flow and pressure in fresh cadavers has greatly enhanced the clinical applicability of cadaveric simulation (7). Nonetheless, limitations with the cadaveric surgical teaching model remain.

Trainees greatly benefit from surgical training in living models that accurately simulate tissue texture, blood circulation, and anatomical structure; practicing among these scenarios prepare trainees to apply these skills in the treatment of human patients (10). Therefore, animal models have been used as an alternative means of surgical training because of their potential to mimic human physiology and pathology. While no model can perfectly replicate the intricacies of human biology, the use of animal models ensures similar insights as with human cadavers into the underlying mechanisms of diseases. For example, the anesthetization of live animals during surgery contributes to the real-life simulation of the operating room, requiring teamwork and enabling the trainees' development of effective intra-operative communication skills. In more recent years, non-animal surgical simulation models have been developed. These deserve consideration as they serve as a companion to animal models in surgical training, or, in some scenarios a more desirable alternative to animal models. In this review, we explore both animal and non-animal surgical simulation approaches that may enhance the surgical training of students and residents, focusing on the fetal and neonatal environments. The potential for various simulation approaches in preparing trainees for urologic surgeries is highlighted.

### The use of animal models for surgical training

Animal models have historically served as indispensable tools in biomedical research, enabling scientists to study diseases, develop treatments, and test hypotheses in a controlled environment. Countless medical breakthroughs, such as the discovery of insulin and the development of vaccines, have been made possible through animal experimentation (11). The use of live animals has been a part of surgical residency training since Halsted introduced it in 1889 (12). A variety of large animals have been used, including swine and dogs. The use of animal models in surgical training has been particularly valuable in fetal or neonatal surgical interventions (13). Animal surgeries offer the opportunity to study long-term outcomes of fetal surgeries, including their effects on fetal growth and development and late-onset complications. Such information can be difficult to obtain from the small numbers of pediatric surgeries in which trainees participate. Moreover, because animal models can be created to mimic various fetal diseases, disorders, and anomalies, the response of the developing fetus to surgical interventions in these scenarios will provide useful information that may be translated to humans.

Surgical training on the fetal and neonatal level is particularly challenging due to several factors. Firstly, fetal and neonatal anatomy and physiology are vastly different from that of adults. Neonatal tissue is friable and delicate. In comparison to adults, physical structures are tiny. The fragility of the tissue and small size of the patient contribute to the complexity of fetal and neonatal surgery, requiring specialized techniques and a high level of precision (14). Secondly, the space in the neonatal chest and abdomen is limited, adding to the technical challenges of surgery. In such a confined surgical space, the surgeon must make many physical and spatial adaptations. The surgeon's development of these skills takes much time and practice (15). Thirdly, successful fetal and neonatal surgeries require a thorough understanding of the unique developmental changes that occur in these early stages of life, which is acquired only via the appropriate combination of didactic and experiential learning. Finally, the combination of underdeveloped organ function and usually life-threatening congenital conditions in fetal surgery leads to significant risks for the fetus, further complicating the surgical process and underscoring the need for adequate training (16). The use of animal models in preparing surgeons for fetal and neonatal surgery could therefore be helpful.

The use of animal models requires careful consideration of the ethical implications. In recent years, these concerns have sparked discussions and debates regarding the necessity of the use of live animals in biomedical research. For example, there is significant pressure on universities by organizations such as People for the Ethical Treatment of Animals to limit any form of animal vivisection (17). Experiments must be designed to minimize animal stress and suffering as much as possible (18). This includes providing appropriate anesthesia and analgesia, housing the animals in humane conditions, and using the minimum number of animals necessary to achieve the research objectives. Research should address important clinical questions and have a reasonable likelihood of leading to improved patient outcomes (19). Rigorous ethical review and approval processes are essential to ensure that animal experiments in this field are justified and conducted in an ethical manner (18).

Finally, there are notable anatomic and physiologic differences from humans. Careful selection of animal models is crucial to obtain meaningful and ethically sound results (20). Differences in anatomy, physiology, and disease progression between species can significantly impact the validity and translatability of the research findings. Accordingly, innovations and other results of surgical intervention in animals may not be immediately applicable to humans and must thus be validated in human trials before being applied to clinical practice. Another drawback of the live animal surgical lab is the complicated infrastructure required to comply with regulations. The cost of maintaining an animal lab for surgical training is also substantial. Whenever feasible, methods that avoid or replace use of animals in research should be used.

### The use of simulation models for surgical training

Surgical simulation offers several benefits, including providing a safe and standardized method for training in surgery without the risks associated with operating on real patients. It allows surgeons to engage in deliberate practices for skills development and refinement. Simulations that challenge trainees to progressively more complex surgical situations can be created, which allows for the training to be adapted to individual trainees. Simulation also offers a secure environment to learn and execute surgical procedures, which improves confidence among individuals and teams and shortens the learning curve (21). Metrics for the standardized assessment of proficiency are easily incorporated into surgical simulation training (22). Additionally, surgical simulation has been shown to reduce error rates committed by residents during their initial surgical procedures (21, 23–25). The models included in our discussion emphasize laparoscopic procedures, where simulation aids in acquiring basic skills and tackling complex surgeries within the constraints of limited anatomical space. Simulation plays a pivotal role in refining skills for delicate urological operations such as hypospadias repair and pyeloplasty. Notably, simulation serves as a vital tool for practicing rare, high-stakes pediatric operations, ensuring surgeons are well-prepared when confronted with such cases.

#### Bench models

The simplest and cheapest form of surgical simulation is the bench model (26). Bench models utilize synthetic materials or non-living animal tissue for the didactic instruction of surgical procedures and the evaluation of technical proficiency during practice. Regulatory and ethical concerns are minimal. There is substantial evidence that bench-top training facilitates skill acquisition (27, 28). For example, the suture tying board is a simple tool for students and residents to practice tying square knots, including in progressively more challenging simulated scenarios. As skills are acquired, learners can practice sewing on Dacron patches and perform end-to-end and end-to-side anastomoses on prosthetic grafts. Laparoscopic box simulators such as the McGill Inanimate System for Training and Evaluation of Laparoscopic Skills (MISTELS) (29) are additional bench model systems that are used to teach and assess basic laparoscopic skills and provide opportunities to learn instrument handling. In the MISTELS and similar simulation systems, trainees hone their skills through exercises such as peg transfer, cutting, suturing, and placing an endoloop (30). As trainees advance, many inanimate models are available for teaching more complex operations. While these models are most useful for the novice surgeon or junior trainee, more advanced models are useful for refining already acquired skills (31).

Pediatric and fetal surgery requires operating in small, confined spaces, which is very different as compared to adults. Given these space constraints, laparoscopic techniques are more challenging for fetal and neonatal surgeons (15). Individual development of these skills can be accomplished through laparoscopic trainers that replicate reduced space conditions and simulate neonatal surgical situations (32). After mastering these basic skills, trainees may use other modalities of simulation to prepare for more complex operative procedures.

#### Virtual reality

Virtual reality (VR) or augmented reality (AR) are the digital recreations of real life (33, 34). In the past few years, this technology has been adapted in medical education (35). VR holds significant promise because it can incorporate unique clinical scenarios. In theory, an infinite number of lessons may thus be designed. For example, with virtual endoscopy simulators, the trainee is taught the basics of performing the procedure with real-time feedback and simulation of the patient's vital signs. Learning how to deal with emergent situations, such as depressed respiratory status from over-sedation, makes the exercise and the test more life-like. Virtual simulators have also been used extensively in teaching laparoscopy, robot-assisted surgery, and endovascular procedures (36). The Robotic Surgery Simulator for the da Vinici robot provides a range of surgical modules encompassing fundamental skills and advanced surgical procedures (37).

Fetal and neonatal surgeries are complex and there is limited case exposure due to the rarity of many pediatric surgical conditions. VR and AR provides practice based on repetition, reducing the learning curve in a safer manner (38). Surgeons and trainees are also able to better understand patient-specific anatomy, anticipate complications, and strengthen teamwork skills (39). These simulators can be useful for multidisciplinary discussion for pre-operative planning approach in complex cases, as evidenced by its use in separation of conjoined twins and planning fetoscopy repair for myelomeningocele (38, 39). Further, these simulators can assist in fostering a better understanding of anatomy and surgery in affected families. Limitations of VR and AR are expense, cybersickness, and the level of fidelity (15).

#### 3-D printing

This technology is just beginning to be used by surgeons to simulate a specific patient's anatomy. We used a 3-D model in our simulation laboratory to create a ureteropelvic junction stenosis (40). We outlined the creation and face validation of a pediatric pyeloplasty simulator, crafted using a cost-effective laparoscopic dry-laboratory model that was developed through 3-D printing and silicone modeling. As the model reaches an appropriate level of realism, there will be further investigation to develop a detailed validation of this pediatric pyeloplasty model as a teaching tool by assessing its education effect on patient outcome (successful pyeloplasty), decreased operative time, and other surgical variables. Combined with patient imaging data, surgeons have the capability to create patient-specific models to provide training and planning tools for complex cases. 3-D printing technology has proved to be extremely helpful in reproducing patient-specific anatomy so that the surgeon can plan the approach (41, 42).

Sandrini et al. showed that models of fetal hearts demonstrate the complexity of both normal and pathological cardiac architecture (43). 3D models can be created with many of the characteristics of the original structures, including their dimensions, geometry, surface roughness and even color (15). 3D models allow surgeons and residents to adopt a more hands-on approach to learning complex procedures without a patient needing to be present (44). This limits risk to patients and allows for more training time. Similar to AR and VR, 3D models can also provide a clearer explanation to parents, facilitating a better understanding of complicated anatomy and intricate procedures (44).

While experts and novices have evaluated usability, realism, and feel of the pyeloplasty model, there is no standardized assessment tool for 3D printed models. There have been efforts to develop a standardized questionnaire to assess the utility of 3D printed models in surgical planning and medical education, drawing input from an expert panel across multiple medical specialties (45). Standardized assessment is essential as 3D printed models become more widely adopted, in order to ensure consistent and reliable evaluation of their educational value and impact across different institutions and contexts.

## Conclusions

Surgical simulation stands at the forefront of surgical education, offering innovative avenues for training future surgeons.

While inanimate bench models are clearly advantageous, they lack the complexity of biological systems. Animal models, both live and cadaveric, as well as human cadavers, offer a more comprehensive and realistic approach to surgical training. However, the ethical and regulatory issues surrounding the use of both necessitate efforts to minimize their use. Despite the importance of advancing non-animal technologies, the current limitations of non-animal alternatives and the ongoing necessity of animal models in certain areas and research should be recognized. By advancing non-animal technologies and responsibly using animal models, surgical education can be further enhanced while upholding ethical standards (21, 46, 47).

Future trends suggest a continued evolution towards high-fidelity simulators capable of replicating entire operations with heightened realism. Patient-specific simulators, mirroring actual anatomy and disease states, are poised to revolutionize practice by allowing surgeons to rehearse specific cases they will encounter. Augmented reality and wireless technologies are paving the way for telesurgery, enabling expert surgeons to remotely guide novices through complex procedures. Validation remains a cornerstone, with the critical need to establish validity before integrating training models into curricula. Various metrics including face, content, construct, concurrent, and predictive validity are vital for comprehensive evaluation (40). Challenges such as subjectivity, lack of gold standards, and resource constraints underscore the complexity of validation efforts. Expanded access to simulation offers trainees opportunities to practice rare procedures in a safe environment conducive to learning from mistakes and honing teamwork skills. Ensuring reproducibility is essential; while low-fidelity simulators provide cost-effective avenues for skill mastery, standardized approaches are needed for consistent reporting of validation methods. In essence, surgical simulation is poised to shape the future of surgical training, emphasizing innovation, robust validation, accessibility, and reproducibility as pillars of its success.
